# Editorial Department

**Published:** 1855-09

**Authors:** 


*Ranking's Abstract*, for July, is received from the publishers, Messrs. S. S. & Wm. Wood, and is for sale at the bookstores. It seems to be a very good number, maintaining its former excellent character as an *omnium gatherum* of the choicer and more valuable portions of our current medical literature.

From the same source we also receive that most valu ed of our exchanges, the British and Foreign Medico-Chirurgical Review. The splendid ability with which the "Review" has been edited, has given it an influence and a name beyond that of any other medical periodical. Dr. Johnson and Forbes, successively, have wielded an immense power over the profession through its pages. For a few years past it has been under the control of Dr. Parkes, who has now gone to Constantinople to assume the supervision of the immense hospital now in process of construction, for the wounded and sick of the Crimea. His place is filled by Dr. E. H. Sieveking, one of the authors of Jones and Sieveking's Pathological Anatomy—at least we judge that Dr. S ieveking is the new editor, from the fact that he furnishes the quarterly report on pathology and medicine, formerly the department of Dr. Parkes.

